# A prognostic Risk Score model for oral squamous cell carcinoma constructed by 6 glycolysis-immune-related genes

**DOI:** 10.1186/s12903-022-02358-0

**Published:** 2022-08-03

**Authors:** Yi Liu, Tong Wang, Ronghua Li

**Affiliations:** Department of Stomatology, Tianjin First Central Hospital, Nankai District, No.24 Fukang Road, Tianjin, 300192 People’s Republic of China

**Keywords:** Oral squamous cell carcinoma (OSCC), Glycolysis, Risk Score, Prognosis, NOMOGRAM

## Abstract

**Background:**

Oral squamous cell carcinoma (OSCC) is the most frequent tumor of the head and neck. The glycolysis-related genes and immune-related genes have been proven prognostic values in various cancers. Our study aimed to test the prognostic value of glycolysis-immune-related genes in OSCC.

**Methods:**

Data of OSCC patients were obtained from the Cancer Genome Atlas (TCGA) and Gene Expression Omnibus (GEO) databases. Enrichment analysis was applied to the glycolysis- and immune-related genes screened by differential expression analysis. Univariate Cox and LASSO Cox analyses were used to filtrate the genes related to the prognosis of OSCC and to construct Risk Score model.

**Results:**

A Risk Score model was constructed by six glycolysis-immune-related genes (including ALDOC, VEGFA, HRG, PADI3, IGSF11 and MIPOL1). High risk OSCC patients (Risk Score >−0.3075) had significantly worse overall survival than that of low risk patients (Risk Score <−0.3075).

**Conclusions:**

The Risk Score model constructed basing on 6 glycolysis-immune-related genes was reliable in stratifying OSCC patients with different prognosis.

**Supplementary Information:**

The online version contains supplementary material available at 10.1186/s12903-022-02358-0.

## Background

Oral squamous cell carcinoma (OSCC) is the most commonly occurred malignancy in the oral cavity [1]. Most OSCCs are correlated with oral precursor lesions [2], and the 5-year overall survival of OSCC patients is less than 40% [3]. Many factors may trigger OSCC, including tobacco smoking, excessive alcohol drinking, chewing betel quid and human papillomavirus (HPV) infection [4]. Moreover, as the most frequent subtype of head and neck squamous cell carcinoma (HNSCC), the metastasis rate of OSCC is high, besides resistance to traditional chemotherapy is usually observed in OSCC patients, leading to undesirable prognosis [5]. Novel biomarkers correlated with the prognosis of OSCC may help to stratify patients with different prognosis and design individual-specific therapy, for example FKBP51 [6] is promising to predict the prognosis of OSCC patients. Whereas, the exploration of more effective and accurate prognostic biomarkers remains a continuous challenge for the medical industry.

Glycolysis, also called Embden-Meyerhof pathway, is an essential metabolic pathway and provides anaerobic energy for body function [7]. In aerobic conditions, pyruvate from glycolysis produces adenosine triphosphate (ATP) for cellular process through oxidative phosphorylation; and in anaerobic conditions, the pyruvate undergoes anaerobic glycolysis [8]. In cells that are not able to generate adequate ATP for body function via oxidative phosphorylation, anaerobic glycolysis may be a way to produce energy [9]. But cancer cells mainly depend on the aerobic glycolysis in order to rapidly provide energy for the tumors even in the presence of sufficient oxygen, which is known as Warburg effect [10]. Therefore, tumor cells intake more glucose to perform aerobic glycolysis. The increased glucose level and the overexpression of glucose transporter proteins (HIF-1α and GLUT-1) are connected with poor prognosis of OSCC [11]. Gong et al. have recently demonstrated that PER1 is a suppressor of glycolysis in OSCC, involving the regulation of cell proliferation [12]. Several aerobic glycolysis related genes have been reported with prognostic or diagnostic values not only in OSCC [13] but also in some other types of cancers, such as breast cancer [14]. Change of ATP supply from oxidative phosphorylation to aerobic glycolysis is thought be the biomarker of T cell activation [15]. The aerobic glycolysis promotes activation of T cells via phosphoinositide 3-kinase (PI3K)/Akt signaling [16]. The activated T cells produce more lactate via increasing lactate dehydrogenase A (LDHA) to support aerobic glycolysis, and increased LDHA is also implicated in poor prognosis of cancer patients [17]. Aerobic glycolysis, a well-known resistance factor for anticancer therapies, is associated with the immunotherapy for cancer patients. Aerobic glycolysis can impact the tumor immunosuppression via a network of pathways in breast cancer [18]. The prognosis of cancers are also closely related to the immune microenvironment and novel prognostic biomarkers for example MIR155HG [19] are reported to have correlation with immune infiltration. However, the potential impacts of glycolysis and immune have been seldom studied in OSCC.

Accordingly, basing on the publicly obtained data, the focus of our work is to discuss the prognostic value of glycolysis-immune-related genes for OSCC patients and to construct a prognostic model using glycolysis-immune-related genes for separating OSCC patients with different prognosis.

## Methods

### Data sources

We downloaded data of 306 OSCC patients, including mRNA expression profiles and corresponding clinical information, and listed the clinical information in Table 1. The data were collected from the Cancer Genome Atlas (TCGA, https://tcga-data.nci.nih.gov/tcga/). Moreover, we downloaded datasets comprised of gene expression profiles and complete survival information of 246 OSCC patients from the Gene Expression Omnibus (GEO, https://www.ncbi.nlm.nih.gov/geo/) database and numbered them GSE85446 (66), GSE65858 (83) and GSE41613 (97). The data of OSCC patients were obtained by the Agilent-014850 Whole Human Genome Microarray 4×44 K G4112F, Illumina HumanHT-12 V4.0 expression beadchip and Affymetrix Human Genome U133 Plus 2.0 Array. Datasets GSE85446, GSE65858, and GSE41613 were merged as meta-GEO dataset, using for subsequent validation.

**Table 1:** Clinicopathological characteristics of OSCC patients from TCGA database

Characteristics		Patients(N = 306)	
		No	%
Gender	Female	102	33.33%
	Male	204	66.67%
Age	≤ 61(Median)	157	51.31%
	> 61(Median)	149	48.69%
Grade	GX	3	0.98%
	G1	49	16.01%
	G2	191	62.42%
	G3	62	20.26%
	Unknown	1	0.33%
Survival time	Long(> 5 years)	31	10.13%
	Short(< 5 years)	275	89.87%
OS status	Dead	143	46.73%
	Alive	163	53.27%

### Differential expression analysis

Limma package [20] in R programming software (version 4.1.0, the same below) was applied to the data we collected above to identify differentially expressed genes (DEGs). The |Log_2_FC|> 0.7 and multiple testing adjusted p value < 0.05 were used as threshold.

### Functional enrichment analysis

The Kyoto Encyclopedia of Genes and Genomes (KEGG) pathways and Gene Ontology (GO) enrichment analyses were applied to the DEGs using “clusterProfiler” package [21] in R programming software. The GO terms and KEGG pathways at the significant level (*p* value < 0.05, adjusted by Benjamini and Hochberg method) were employed.

### Cluster analysis and calculation of ImmuneScore

The mRNA expression profiles were subjected to cluster analysis based on the “K-mean” method utilizing R programming software. The ImmuneScore of each patient was calculated by the “ESTIMATE” function.

### LASSO Cox regression analysis

Univariate Cox analysis was applied to expression profiles of the OSCC patients based on the expression values of selected genes. To screen the genes which were significantly associated with the overall survival (OS) of OSCC, *p* value < 0.01 was used as threshold. To further optimize the genes significantly associated with OS of OSCC, LASSO Cox regression analysis was performed on the screen DEGs using glmnet package [22] in R programming software. Risk Score of each patient was calculated by the following formula:

$${\text{Risk Score}} = \sum_{{{\text{i}} = {1}}}^{{\text{n}}} {\text{Coef}}_{{\text{i}}} {\text{*X}}_{{\text{i}}} {,}$$

Coefi was the LASSO Cox risk coefficient and Xi was the expression value of genes (mRNA expression in this research). Risk Score was tested using the survival, survminer and two-sided log-rank test in R programming software. Then patients were assigned into high-risk and low-risk groups according to the median of Risk Score.

### Kaplan–Meier survival analysis

OS rates of OSCC patients were estimated using the survival and survminer packages in R programming software. The significance of difference of OS rates between high- and low-risk groups was tested by the log-rank or breslow test. Multivariate Cox regression model was used to analyze the independence of the prognostic value of Risk Score.

### Proportion of immune cell infiltration

We used “CIBERSORT” [23] software to estimate the infiltrations of immune cells for OSCC patients. The “CIBERSORT” software, utilizing the deconvolution algorithm, was based on the gene expression matrix. And it characterized the composition of the immune cells by the predisposed 547 barcode genes. The sum of the estimated proportion of the 22 immune cells was one. The significance of difference of immune infiltration ratios was tested by the wilcoxn method.

### Construction of nomogram model

We used rms package in R programming software to construct a prognostic nomogram model by using all independent prognostic factors examined by multivariate Cox regression model and examined the efficiency of the nomogram model by drawing a calibration curve of nomogram.

## Results

### Patients with different prognosis defined by glycolysis-immune-related genes

**Fig. 1:**
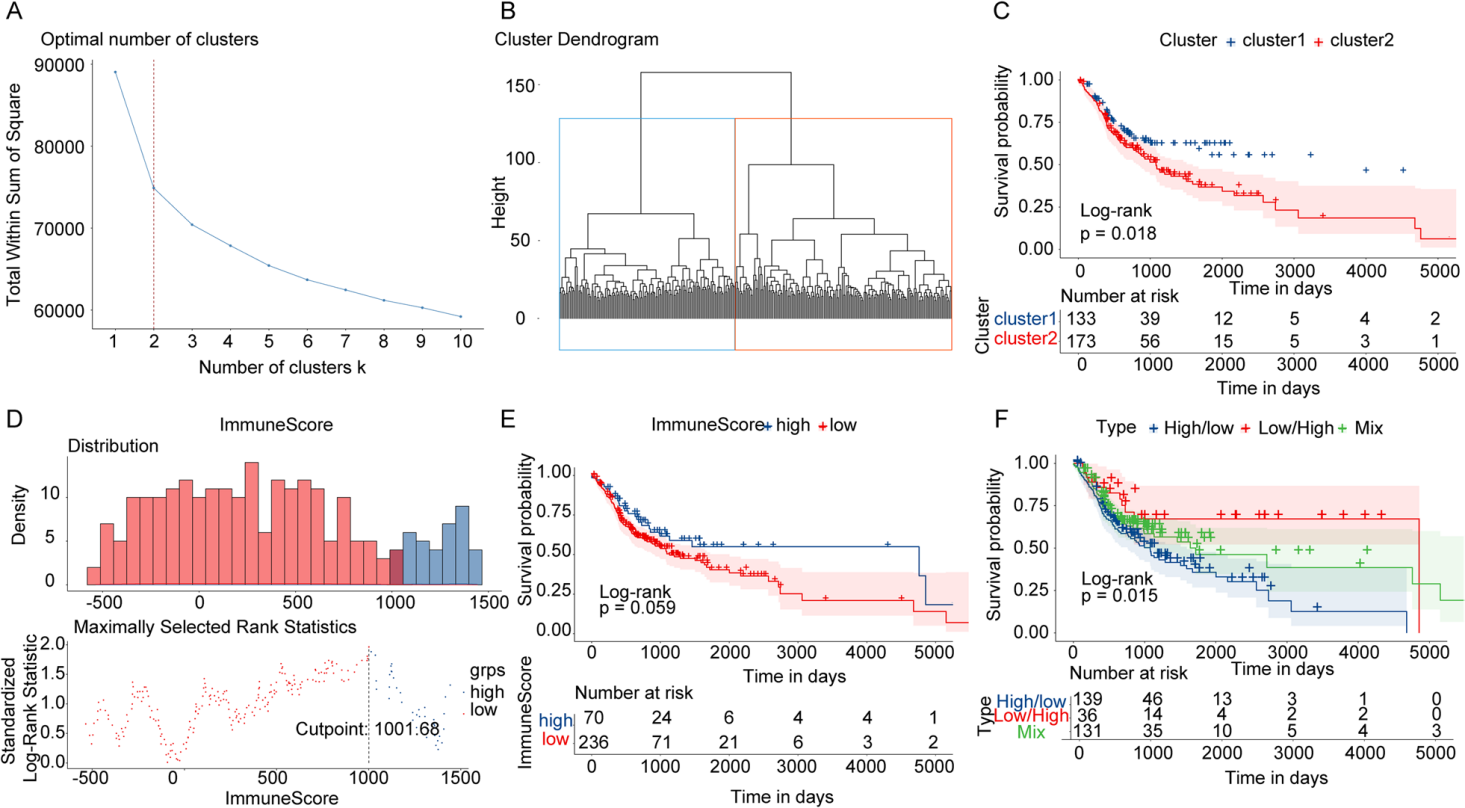
Different prognosis defined by glycolysis in combination with immunity. **A** The elbow figure to determine the number of clusters. The x-axis and y-axis are the number of cluster K and the sum of the square errors. K = 2 is determined by the slope of the curve. **B** Clustering sketch of patients. Different colors represent different clusters. **C** The Kaplan–Meier survival curves defined by glycolysis. The x-axis and y-axis are time and survival probability. The blue is cluster1 (low-glycolysis), and the red is cluster 2 (high-glycolysis). The p value is calculated by the log-rank test. **D** The best cutoff value of the two groups defined by ImmuneScore. **E** The Kaplan–Meier survival curves defined by ImmuneScore. The x-axis and y-axis are time and survival probability. The blue is high-ImmuneScore, and the red is low-ImmuneScore. **F** The Kaplan–Meier survival curves defined by glycolysis in combination with ImmuneScore. The x-axis and y-axis are time and survival probability. The blue is High/Low, the red is Low/High, and the green is the Mix group

Cluster analysis was applied to the patients’ mRNA expression profiles utilizing the 296 glycolysis-related genes downloaded from the GSEA database (Additional file 1: Table S1). According to the sum of the square errors (SSE) (Fig. 1A), the patients were divided into 2 clusters (k = 2) (Fig. 1B). The OS between cluster1 and cluster2 differed significantly via Kaplan–Meier survival analysis (Fig. 1C). The prognosis of cluster1 (low-glycolysis group) was significantly better than cluster2 (high-glycolysis group) (*p* = 0.018).

Then the patients were grouped into high-ImmuneScore group (ImmuneScore > 1001.68) and low-ImmuneScore group (ImmuneScore < 1001.68) according to the cutoff value (Fig. 1D). Survival analysis results showed that the survival probability of the low-ImmuneScore group was lower than that of the high-ImmuneScore group (Fig. 1E) (*p* = 0.059).

Then the patients were reassigned into low-glycolysis-high-ImmuneScore (Low/High) group, high-glycolysis-low-ImmuneScore (High/Low) group and Mix group according to the ImmuneScore and glycolysis. The prognosis of Low/High was better than that of the High/Low group while the prognosis of Mix group was in the middle of the two groups and the prognosis of the three groups differed significantly (Fig. 1F) (*p* = 0.015).

### Screening of glycolysis-immune-related genes

**Fig. 2:**
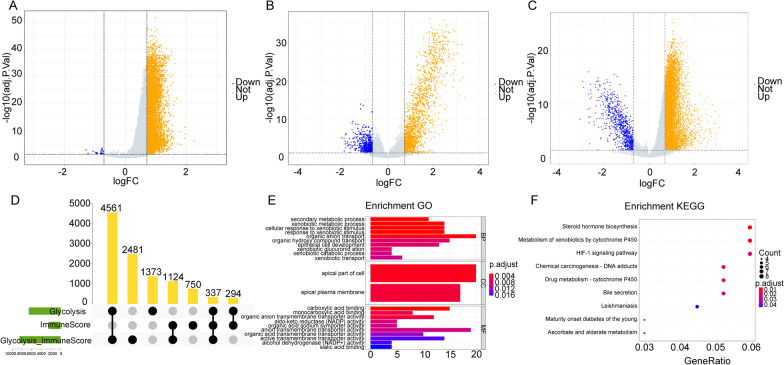
Results of differential analysis and enrichment analysis. **A–C** The volcano plot of DEGs in different groups. The x-axis and y-axis are the multiple of differential expression (log_2_FC) and − log_10_(adj. p. val). The blue and orange represent downregulation and upregulation genes. **D** The figure containing all DEGs. **E** The significantly enriched GO terms. The x-axis is the number of enriched genes and y-axis is the names of the GO terms. **F** The significantly enriched KEGG pathways. The x-axis is the number of enriched genes and y-axis is the names of the KEGG pathways

Differential expression analysis was applied to the expression data of OSCC patients to screen the differentially expressed genes (DEGs). There were 2505 DEGs (Fig. 2A) between the low-glycolysis and high-glycolysis groups, 6565 DEGs (Fig. 2B) between low-ImmuneScore group and high-ImmuneScore group and 8503 DEGs (Fig. 2C) between the Low/High and High/Low group. And 337 overlap genes among the three sets of DEGs (Additional file 2: Table S2, Fig. 2D) were found.

Enrichment analysis was performed on the 337 overlap genes. The enriched GO terms and KEGG pathways were listed in Additional file 3: Table S3 (the pathways were obtained basing on KEGG [24–26]). The significantly enriched GO terms and KEGG pathways were shown on Fig. 2E and F. The results demonstrated that the 337 overlap genes were significantly enriched in the secondary metabolic process, apical part of cell and carboxylic acid binding GO terms, as well as steroid hormone biosynthesis and HIF-1 signaling KEGG pathways. The genes were significantly enriched in the pathways which were associated with metabolism, inflammation and cancers [27–30].

### Construction and validation of prognostic model

**Fig. 3:**
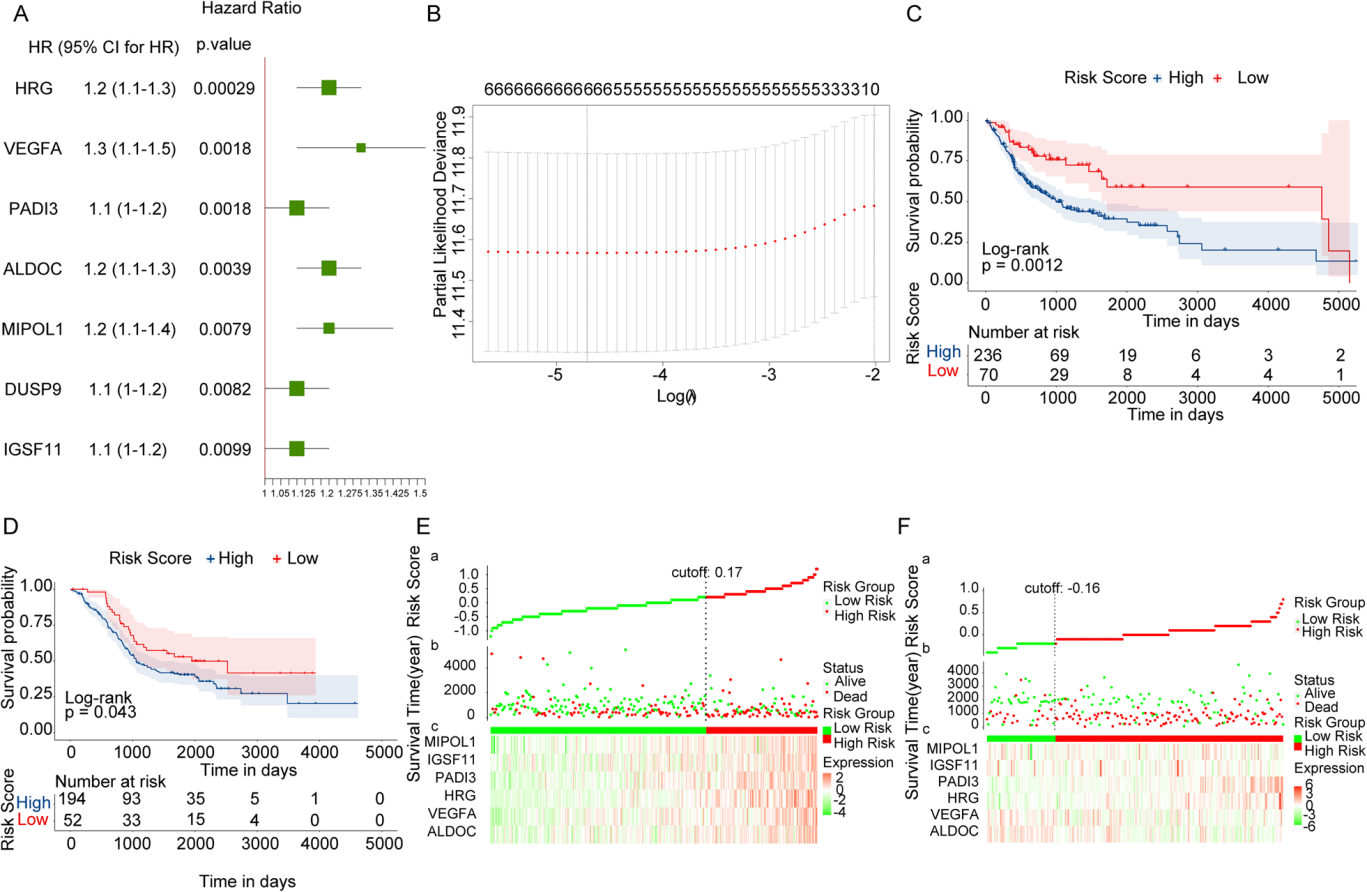
Construction of OSCC prognostic model. **A** The forest plot of seven genes significantly associated with the prognosis of OSCC by univariate Cox regression analysis. HR is short for Hazard ratio and 95%CI is 95% of the confidence interval. **B** The graph to determine the best lambda by LASSO Cox model. The x-axis is the log (lambda) and y-axis is the partial likelihood deviance whose smallest value is the best lambda value. **C** The Kaplan–Meier survival curve in TCGA dataset. The x-axis is time and y-axis is the survival probability. The red is the low-risk group and the blue is the high-risk group. *p* value is calculated by the log-rank test. **D** The Kaplan–Meier survival curve in meta-GEO dataset. The x-axis is time and y-axis is the survival probability. The red is the low-risk group and the blue is the high-risk group. *p* value is calculated by the log-rank test. **E** and **F** The estimated Risk Score of each patient is ranked from small to big. The vertical dotted line is the median of Risk Score

In the TCGA dataset, 337 overlap genes were used as continuous variable to perform univariate Cox regression analysis. Hazard ratio (HR) of each gene was calculated and *p* < 0.01 was used as threshold to screen the genes which were associated with OS of OSCC (Fig. 3A). Seven genes were selected and then LASSO Cox regression analysis was applied to the 7 genes to screen a set of genes which were significantly associated with the prognosis of OSCC. Six genes (including ALDOC, VEGFA, HRG, PADI3 IGSF11 and MIPOL1) were screened according to the lowest lambda value (Fig. 3B).

In order to obtain a uniform critical value, we standardized the the 6 gene expression values in the TCGA and three GEO datasets to a value with median 0 and standard deviation 1. Then the standardized expression values were multiplying regression coefficient to construct a Risk Score model as follows: Risk Score = (0.039770315*ALDOC) + (0.164774576*VEGFA) + (0.208429564*HRG) + (0.175124998*PADI3) + (0.065430137*IGSF11) + (0.001152926*MIPOL1). We calculated Risk Score of each patient. And patients in the TCGA and meta-GEO datasets were assigned into high-risk and low-risk groups according to the best cutoff value of Risk Score (− 0.3075). OS of patients in the high-risk group was lower than that in the low-risk group both in the TCGA and meta-GEO datasets (including GSE85446, GSE65858 and GSE41613) from the survival analysis (Fig. 3C–D).

According to the survival time in combination with patients which were ranked by the Risk Score in TCGA and meta-GEO datasets, there were more patients in the high-risk group compared to the low-risk group. In Fig. 3E and F, the green dots and red dots represent alive and dead patients. And the number of dead patients in the high-risk group was higher than that in the low-risk group.

In conclusion, the results suggested that Risk Score model constructed by ALDOC, VEGFA, HRG, PADI3 IGSF11 and MIPOL1 might be a reliable prognostic model.

### Risk Score as an independent prognostic hallmark of OSCC

**Fig. 4:**
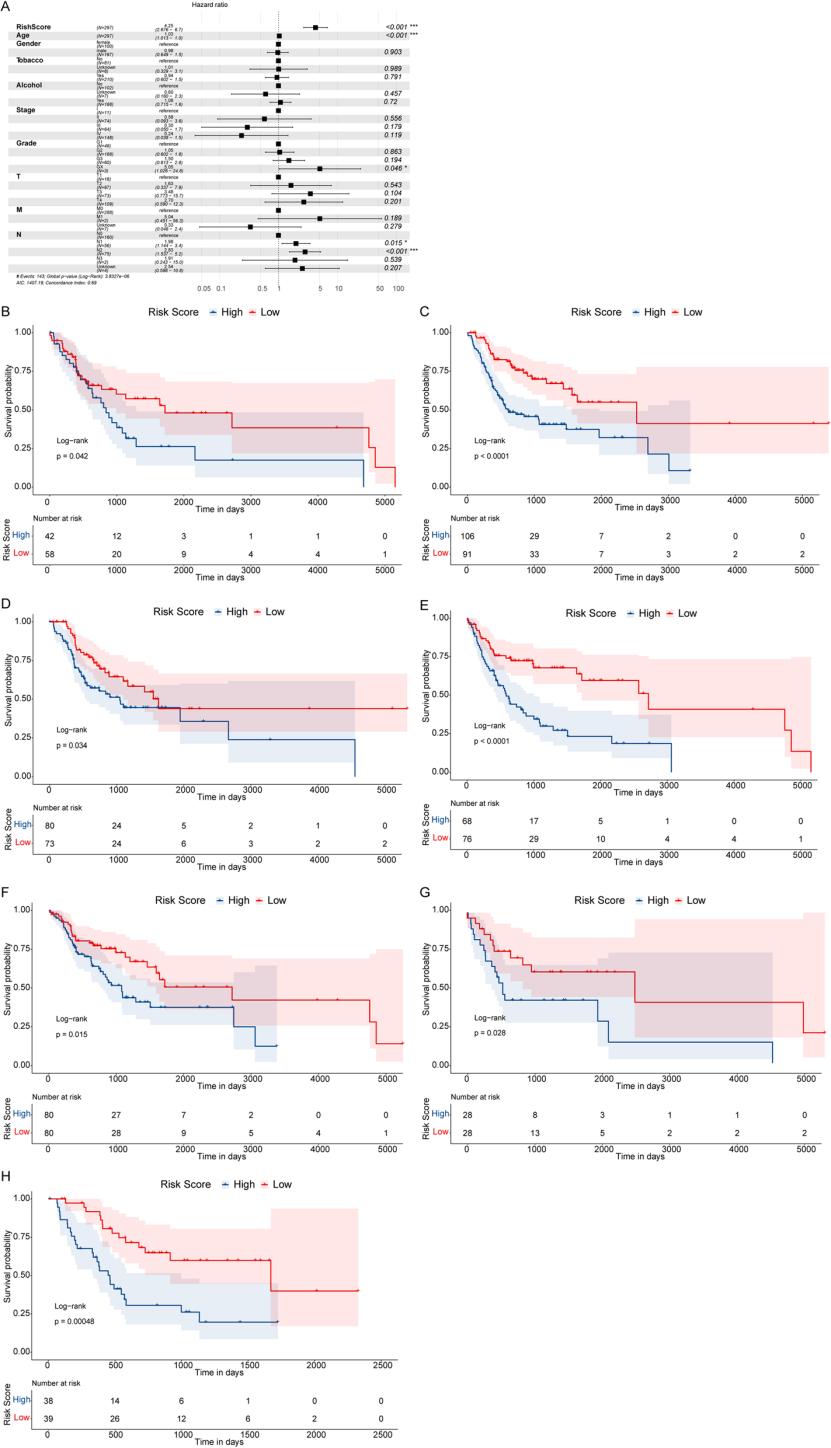
Risk Score is an independent prognostic biomarker for OSCC. **A** Forest plot of multiple Cox regression analysis. Compared to the reference, patients with Hazard ratio > 1 were considered higher death risk, and patients with Hazard ratio < 1 were considered lower death risk. **B**, **C** Kaplan–Meier survival curves in female and male subgroups, separately. **D**–**E** Kaplan–Meier survival curves of ≤ 61-year old and > 61-year old subgroups, separately. **F**–**H** Kaplan–Meier survival curves of patients in N0, N1, N2-N3 subgroups, separately

Multivariate Cox regression analysis was then conducted including totally 10 factors, comprising Risk Score, age, gender, Grade, tobacco history, alcohol history, tumor stage, TNM status, to determine the independent prognostic indicators for OSCC patients (Fig. 4A) (removing one non-differentiated sample and 8 samples without stage & T status information). The results showed that the Risk Score, age, Grade, and Node status were significantly correlated with the OS of OSCC (Fig. 4A). Patients in the low-risk group had lower death risk and Risk Score was a reliable prognostic factor (HR = 4.25, 95%CI: 2.676—6.7, *p* < 0.001).

To further explore the prognostic values of Risk Score in different pathological factors such as age, gender, and Node status, we regrouped the patients to perform Kaplan–Meier survival analysis. Our data suggested that high risk OSCC patients in various subgroups all had inferior survival compared to the patients in low-risk group, including female patients (Fig. 4B), male patients (Fig. 4C), younger patients (≤ 61-year old) (Fig. 4D), older patients (> 61-year old) (Fig. 4E), and N0, N1, N2-N3 patients (Fig. 4F-H). Above results indicated that the Risk Score was an independent prognostic indicator for stratifying the OSCC patients with different prognosis.

### Nomogram model predicts the survival probability of OSCC patients with good performance

**Fig. 5:**
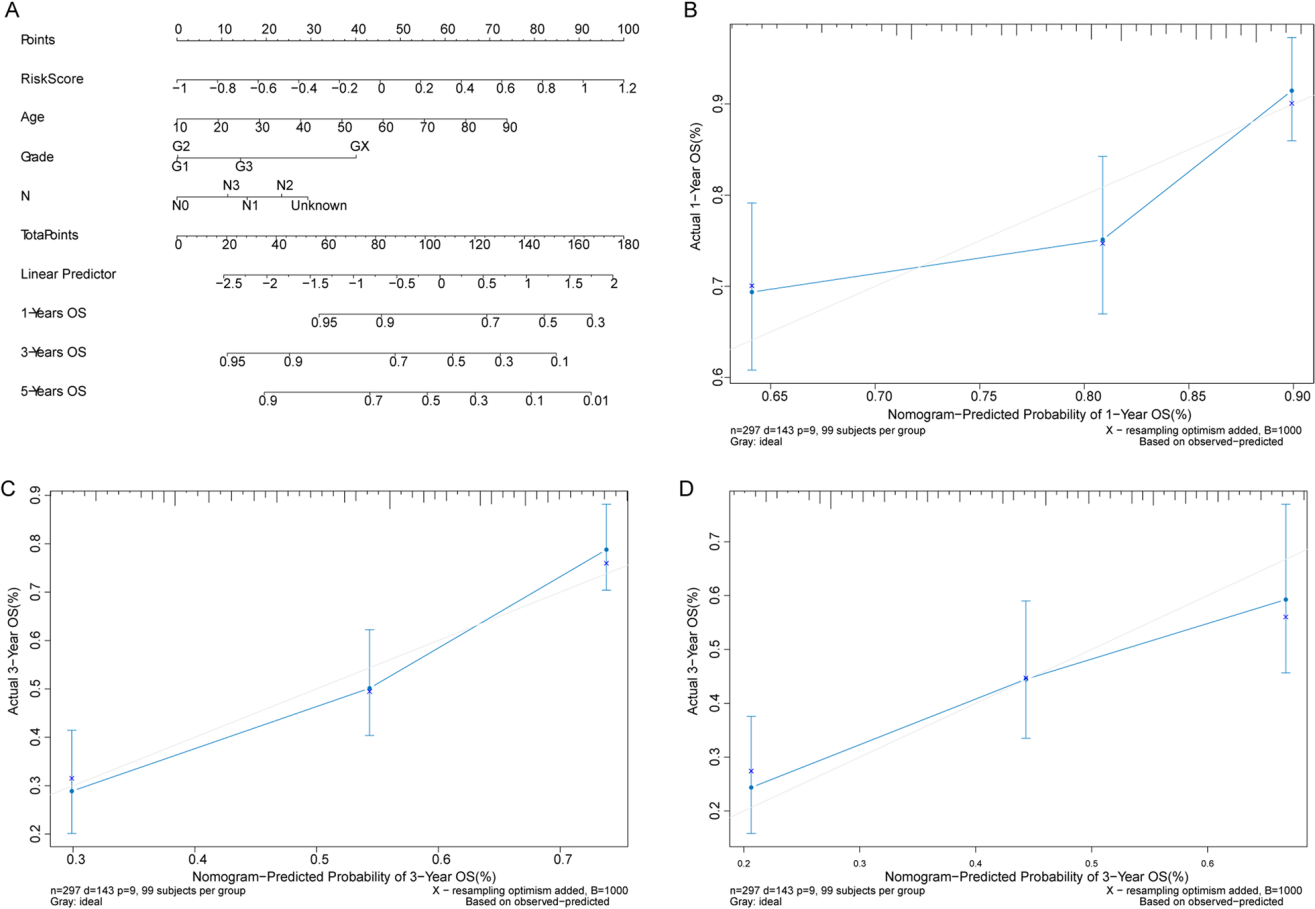
Nomogram model to predict the prognosis of OSCC patients. **A** Nomogram to the living probability of OSCC patients in 1 year, 3 years and 5 years. **B**–**D** The calibration curve of nomogram for OSCC patients in 1 year, 3 years and 5 years, respectively. The x-axis is the survival probability estimated by nomogram and y-axis is the actual survival probability

Subsequently, the independent prognostic factors, Risk Score, age, Grade, and Node status, were used to construct the nomogram model (Fig. 5A) to predict the survival probability of OSCC patients in 1 year, 3 years and 5 years. In the calibration graph, the adjusted curves were close to the ideal curve (a line through the origin with the slop 1 and 45 degree). These indicated that the estimated survival probability agreed well with the actually living probability (Fig. 5B–D) in 1 year, 3 years and 5 years.

### The immunosuppressive cells of OSCC patients were significantly infiltrated in the low-risk group

**Fig. 6:**
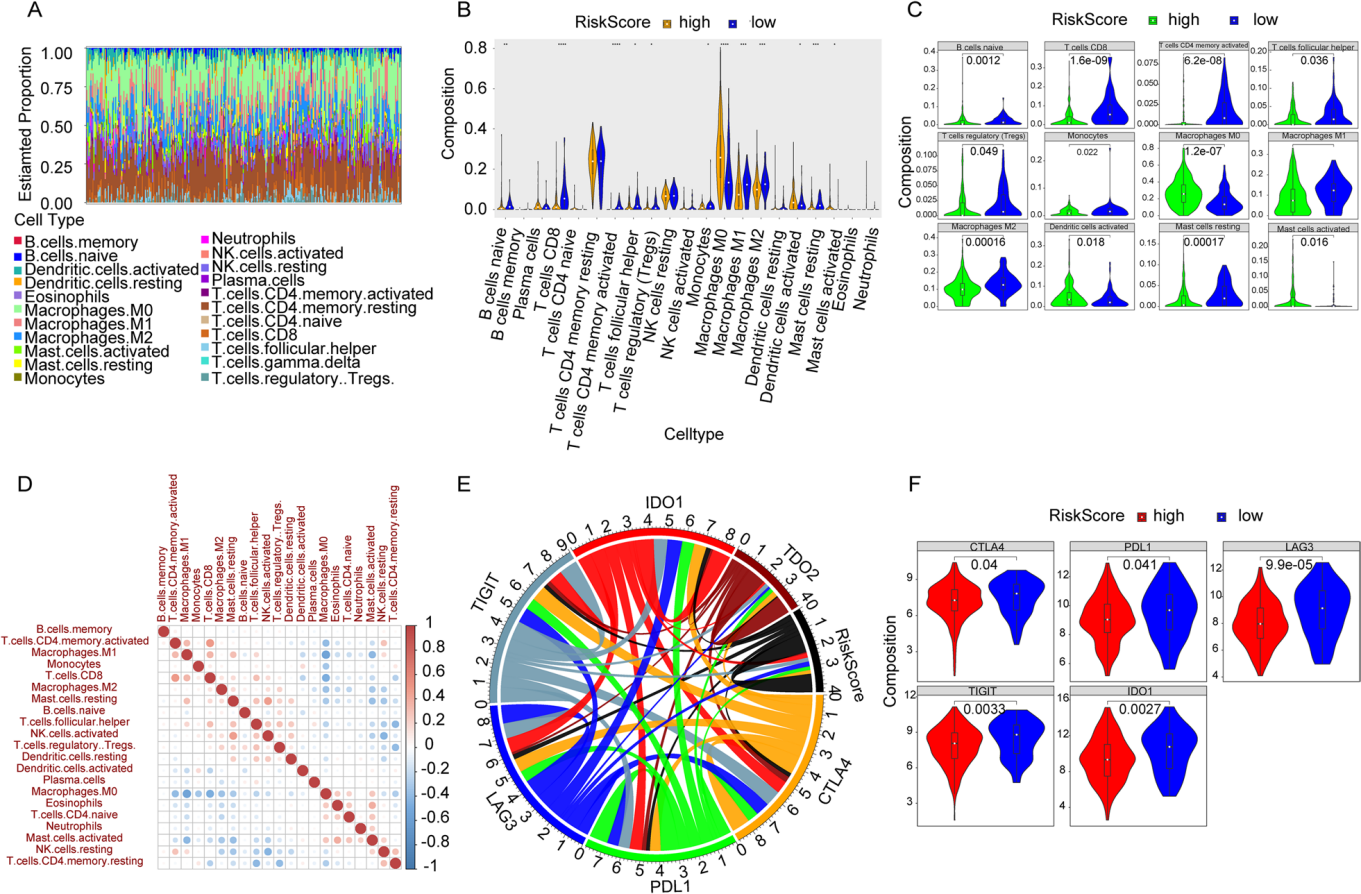
Immune infiltration between the high- and low-risk groups. **A** The relative infiltration ratio of immune cells in all OSCC patients. **B** The violin diagram of immune cells of the difference between the high- and low-risk groups. The x-axis is the 21 immune cells and the y-axis is the infiltration ration of immune cells. We used the wilcoxn method to calculate the p value. (ns: *p* > 0.05, *: *p* <  = 0.05, **: *p* <  = 0.01, ***: *p* <  = 0.001, ****: *p* <  = 0.0001). **C** The box graph of differentially expressed immune cells between the high- and low-risk groups. The x-axis is the high- and low-risk groups. The y-axis is the infiltration ratio of immune cells and *p* value is calculated by the wilcoxn method. **D** The correlation matrix of 22 immune cells. The orange is positive correlation and the blue represents negative correlation. The greatness of the correlation is positively related to the darkness of the color. **E** The chord diagram of Risk Score and 5 immune checkpoints. The width of the connecting lines represent the strength of the correlation. **F** The violin diagram of the difference of expression of the checkpoints between the high- and low-risk groups. The red is high-risk group and the blue is low-risk group. The y-axis is the expression of the checkpoints, and p value is calculated by the wilcoxn method

After organizing the immune infiltrations of 306 OSCC patients (Fig. 6A), we could find that the proportions of the immune infiltration between the high- and low-risk groups differed significantly (Fig. 6B). The infiltration ratios of B cells naive, T cells CD8, T cells CD4 memory activated, T cells follicular helper, T cells regulatory, monocytes, macrophages M0, macrophages M1, macrophages M2, dendritic cells activated, mast cells testing and mast cells activated differed significantly (Fig. 6C). The infiltration ratios of B cells naive, T cells CD8, T cells CD4 memory activated, T cells follicular helper, T cells regulatory, monocytes, macrophages M1, macrophages M2 and mast cells testing were higher in the low-risk group, while the infiltration ratios of macrophages M0, dendritic cells activated and mast cells activated were higher in the high-risk group. The correlation of different immune cells was relatively weak, which might imply the weak interactions among immune cells in OSCC (Fig. 6D).

We analyzed the correlation between the Risk Score and the immune checkpoints (CTLA4, PDL1, LAG3, TIGIT, IDO1 and TDO2), and the results showed that the Risk Score related to 6 immune checkpoints (Fig. 6E). The expressions of LAG3 (*p* = 9.9*e^−5^), TIGIT (*p* = 0.0033) and IDO1 (*p* = 0.0027) were significantly higher in the low-risk group compared to the high-risk group (Fig. 6F). Moreover, higher PDL1 expression was observed in low-risk OSCC patients, and similar PDL1 expression tendency had also been documented in previous reports [31, 32].

## Discussion

OSCC has been widely considered the most frequent malignancy of the head and neck, novel prognostic biomarkers for OSCC are helpful for stratifying patients with different prognosis [33]. In this study, basing on the public OSCC data, glycolysis-immune related genes were employed to build predictive prognostic model for OSCC. Our Risk Score, based on ALDOC, VEGFA, HRG, PADI3, IGSF11 and MIPOL1, was a promising prognostic indicator for OSCC.

Firstly, we downloaded data of OSCC patients from public databases to perform differential expression analysis and functional enrichment analysis. There were 337 overlap DEGs among the groups defined by glycolysis and ImmuneScore, and these genes were enriched in 99 GO terms and 9 KEGG pathways. We noticed that several metabolism related terms were significantly enriched, including secondary metabolic process, apical part of cell and carboxylic acid binding GO terms and the steroid hormone biosynthesis pathway. The carboxylic acid is an important substance in the generation and progress of cancers [34]. Besides, the biosynthesis of steroid hormone, a process requiring multiple enzymes to coordinate [35], is also found to be related to the formation and growth of prostate cancer [36]. Whereas, their roles in OSCC have remained largely unclear. Additionally, the HIF-1 signaling pathway was also significantly enriched. Hypoxia is a common feature of many tumors. Under hypoxia environment, HIF-1 is able to bind to the hypoxia response elements (HREs) of target genes, and the target genes included the genes encoding glycolytic receptors and enzymes [37]. Thus, HIF-1 signaling pathway might imply the indirect correlation between hypoxia and our Risk Score, whose details deserve further investigation.

Then we optimized the DEGs using univariate Cox and LASSO Cox regression analysis and 6 genes were selected, including ALDOC, VEGFA, HRG, PADI3, IGSF11 and MIPOL1. Previous studies have shown that the ALDOC is a member of the aldolase family and has been identified as an independent prognostic hallmark for cancers [38]. Besides, ALDOC has been evidenced to inhibit the migration of OSCC and serve as a prognosis marker [39], which was in line with our data. The VEGFA is responsible for the formation of new blood vessels, which is important for the progression of cancers [40]. VEGFA has also been reported as single gene prognosis marker in OSCC [41], while we firstly included it in the glycolysis-immune related prognostic Risk Score. The HRG expression is connected with prognosis of colorectal cancer [42]. The HRG has been used as prognostic biomarker for many kinds of cancers such as prostate cancer [43]. However, few HRG related studies were found in OSCC. The PADI3 has anticancer effect through the arresting of cell cycle in colon cancer, and it regulates the glycolysis in multiple cancer cell types [44]. The MIPOL1 may induce tumor suppression in nasopharyngeal carcinoma resulting anticancer effect [45]. Although two genes (HRG and PADI3) have been seldom studied in OSCC or HNSCC, their roles in other tumors could be found, indicating that more related exploration should be done in OSCC. The above evidence indicated that these genes, which were associated with glycolysis and immunity, are related to progression of cancers, which suggesting that the glycolysis-immune-related genes might be reliable prognostic biomarkers for OSCC patients.

Our prognostic Risk Score was constructed basing on the 6 core genes to separate patients with different prognosis, and patients in the TCGA and GEO databases were assigned to high- and low-risk groups according to the best cutoff of Risk Score. The Kaplan–Meier survival demonstrated that patients in the high-risk group had lower survival rates than those in the low-risk group, indicating the relatively reliable performance of the Risk Score. The Risk Score models constructed by glycolysis-related genes have been proven prognostic values in pancreatic ductal adenocarcinoma [46]. Prognostic models basing on immune-related genes have also been identified in renal papillary cell carcinoma [47]. The prognostic models constructed by glycolysis-related genes or immune-related genes have been proven reliable in many types of cancers, which are in accord with our results. Additionally, between high and low risk OSCC patients, 12 types of immune cells were significantly differentially infiltrated, indicating the different immune microenvironment between the patients with different prognosis. Herein, relatively higher abundance of multiple immune cells were observed in low risk patients, including Treg and Macrophages M2 cells. Tregs have been reported to inhibit the antigen-presenting cells and thereby promote the proliferation of tumor cells, meanwhile Tregs are also related to undesirable prognosis [48]. However, more recently, it has been indicated that the infiltration, activation, and survival of Tregs involve in complicated multiple processes in TME, which differ among distinct tumor types [48]. Moreover, complicated factors, including the composition and activity of infiltrated immune cells in tumor immune microenvironment and the cell surface expression of immune checkpoints, together determine the immune response states in tumor microenvironment [49, 50] Several key immune checkpoints also showed distinct expression between high and low risk OSCC patients. To the best of our knowledge, this is the first study assessing the prognostic value of glycolysis-immune-related genes for OSCC patients, known to be implicated in stratifying patients with different prognosis.

Nevertheless, some limitations of our study were needed to be further improved in the near future. Although we have validated our prognostic Risk Score in TCGA and meta-GEO datasets, more validation in the expanded sample size might further elevate the confidence of the Risk Score. Additionally, more underlying functional details about the 6 core genes of Risk Score in OSCC should be investigated in our future work.

## Conclusions

In this work, we have firstly revealed a reliable glycolysis-immune related prognostic Risk Score for OSCC patients, basing on multiple OSCC public datasets. The prognostic Risk Score was based on 6 core genes, comprising ALDOC, VEGFA, HRG, PADI3, IGSF11 and MIPOL1. Our prognostic Risk Score model might be helpful in separating OSCC patients with different prognosis, shedding new light on the future management choosing of OSCC patients.

## Supplementary Information

**Additional file 1**.** Table S1**. The glycolysis-related genes from GSEA database.

**Additional file 2**.** Table S2**. The overlap genes of the DEGs screened by levels of glycolysis and Immune Score.

**Additional file 3**.** Table S3**. The enriched GO terms and KEGG pathways.

## Data Availability

The datasets analysed during the current study are available in The Cancer Genome Atlas (TCGA, https://tcga-data.nci.nih.gov/tcga/) and the Gene Expression Omnibus (GEO, https://www.ncbi.nlm.nih.gov/geo/, accession number: GSE85446, GSE65858 and GSE41613) databases.

## Abbreviations

ATP: Adenosine triphosphate
DEGs: Differentially expressed genes
GEO: Gene expression omnibus
GO: Gene ontology
HR: Hazard ratio
HPV: Human papillomavirus
LDHA: Lactate dehydrogenase A
KEGG: Kyoto encyclopedia of genes and genomes
OSCC: ORAL squamous cell carcinoma
OS: Overall survival
SSE: Sum of the square errors
TCGA: The cancer genome atlas
